# Distinguishing motion artifacts during optical fiber-based *in-vivo* hemodynamics recordings from brain regions of freely moving rodents

**DOI:** 10.1117/1.NPh.11.S1.S11511

**Published:** 2024-05-24

**Authors:** Anupam Bisht, Kathryn Simone, Jaideep S. Bains, Kartikeya Murari

**Affiliations:** a University of Calgary, Biomedical Engineering Graduate Program, Calgary, Alberta, Canada; b University of Calgary, Hotchkiss Brain Institute, Calgary, Alberta, Canada; c University of Calgary, Cumming School of Medicine, Department of Physiology and Pharmacology, Calgary, Alberta, Canada; d University of Calgary, Electrical and Software Engineering, Calgary, Alberta, Canada

**Keywords:** motion artifacts, *in-vivo* fiber photometry, hemodynamics, intrinsic optical imaging, Monte Carlo simulation, oxygen saturation

## Abstract

**Significance:**

Motion artifacts in the signals recorded during optical fiber-based measurements can lead to misinterpretation of data. In this work, we address this problem during *in-vivo* rodent experiments and develop a motion artifacts correction (MAC) algorithm for single-fiber system (SFS) hemodynamics measurements from the brains of rodents.

**Aim:**

(i) To distinguish the effect of motion artifacts in the SFS signals. (ii) Develop a MAC algorithm by combining information from the experiments and simulations and validate it.

**Approach:**

Monte-Carlo (MC) simulations were performed across 450 to 790 nm to identify wavelengths where the reflectance is least sensitive to blood absorption-based changes. This wavelength region is then used to develop a quantitative metric to measure motion artifacts, termed the dissimilarity metric (DM). We used MC simulations to mimic artifacts seen during experiments. Further, we developed a mathematical model describing light intensity at various optical interfaces. Finally, an MAC algorithm was formulated and validated using simulation and experimental data.

**Results:**

We found that the 670 to 680 nm wavelength region is relatively less sensitive to blood absorption. The standard deviation of DM ($\sigma_{DM}$) can measure the relative magnitude of motion artifacts in the SFS signals. The artifacts cause rapid shifts in the reflectance data that can be modeled as transmission changes in the optical lightpath. The changes observed during the experiment were found to be in agreement to those obtained from MC simulations. The mathematical model developed to model transmission changes to represent motion artifacts was extended to an MAC algorithm. The MAC algorithm was validated using simulations and experimental data.

**Conclusions:**

We distinguished motion artifacts from SFS signals during in vivo hemodynamic monitoring experiments. From simulation and experimental data, we showed that motion artifacts can be modeled as transmission changes. The developed MAC algorithm was shown to minimize artifactual variations in both simulation and experimental data.

## 1 Introduction

Hemodynamic changes in the brain are closely coupled with neuronal activity by a relationship referred to as neurovascular coupling (NVC).^1^^,^^2^ Studying NVC is important due to its impact on neurological disorders as well as to understand its mechanisms.^2^^,^^3^ Hemodynamic recordings from the brain can be performed through many modalities such as widefield systems, head-mountable microscopes, and fiber-based instruments.^4^ The latter are minimally invasive and enable deep brain investigations during preclinical and clinical studies.^5^^–^^9^ During these fiber-based recordings, one or more fibers are used to deliver and collect narrowband or broadband light from the sample of interest, e.g., tissue. The collected light signals are then processed to extract the optical properties of the tissue sample.^10^

Motion artifacts during fiber-based recordings have been reported to give rise to problems such as changes in the transmitted/collected light leading to misinterpretation of data.^6^^,^^11^^–^^13^ A limited number of strategies to mitigate motion artifacts from signals acquired from fiber-based instruments have been previously implemented. These include trial rejection, development of instrument design for rejecting motion artifacts, and trial averaging.^14^ Motion artifact correction (MAC) algorithms have been developed for near-infrared spectroscopy (NIRS) and fiber-based fluorescence measurements.^13^^,^^15^ Motion artifacts in the widely used gCaMP-based fluorescence fiber photometry for monitoring intracellular calcium (${\mathrm{Ca}}^{2+}$) are estimated and corrected using ∼405 to 410 nm light, which is an isosbestic wavelength of the fluorophor that is responsive only to the total concentration and does not vary with ${\mathrm{Ca}}^{2+}$ concentration.^13^ This isosbestic channel removes the artifacts present in the 465 nm fluorescence excitation channel. During NIRS measurements, the motion of the scalp relative to the fiber results in motion artifacts.^14^ Signal processing methods, such as principal component analysis, and spline interpolation, have been established to correct for motion artifacts in NIRS signals.^15^ However, these techniques are mostly modality/paradigm specific.

In this work, we investigate motion artifacts during recordings from a class of optical fiber-based instruments known as single fiber systems (SFS). Development of SFS for diffuse optical measurements has been previously reported in literature.^6^^,^^8^^,^^16^^–^^18^ Our research group has established the use of an SFS to enable hyperspectral reflectance recordings from deep brain regions of freely moving rodents.^6^^,^^17^ It has been shown to reliably measure changes in oxygen saturation (% ${\mathrm{sO}}_{2}$) and blood perfusion changes in the past.^6^^,^^17^^,^^19^ Data recorded using SFS showed an increase in variations in the % ${\mathrm{sO}}_{2}$ as a mouse transitioned from the anesthetized state to a freely moving state.^6^ It was unclear whether the observed variations were part of hemodynamics or due to noise, such as motion artifacts.

In this work, first, we identify a wavelength region in the reflectance data acquired by the SFS that is less sensitive to blood absorption-induced changes (i.e., hemodynamics). Using this wavelength region, we developed a dissimilarity metric (DM) to quantify the effect of motion artifacts during animal experiments. Animal experiments were performed specifically to produce episodes of large motion during SFS recordings from a deep brain region known as the periventricular nucleus (PVN) of the hypothalamus. In experiment-1, we transitioned a mouse between two arenas, which is a scenario representative of spontaneous brain activity and large motion artifacts during transfer. Footshocks (FS) are known to increase brain activity in multiple brain regions including the PVN, while leading to jumping behavior due to short periods of panic.^20^^,^^21^ In experiment-2, FS were applied to the animal, which is representative of a scenario of large motion artifacts during FS due to jumping behavior. We used a combination of experimental data and Monte Carlo (MC) simulations to develop a mathematical modelling framework for the motion artifacts in the SFS signals. This framework was then extended to a MAC algorithm that was validated on simulation and experimental data.

## 2 Methods

**Fig. 1:**
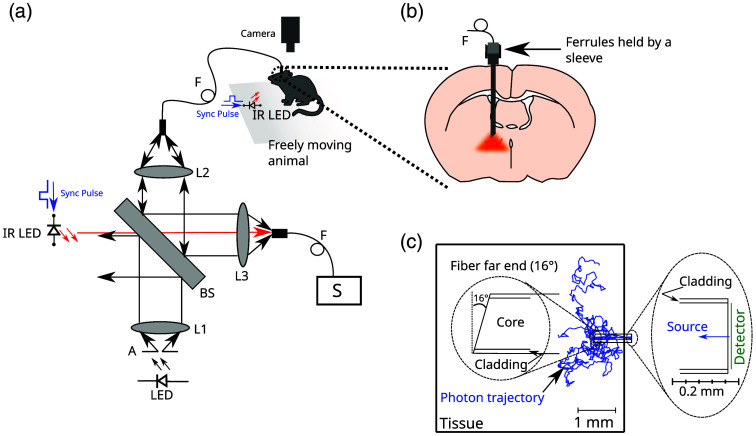
(a) Schematic of the SFS showing optical elements - lenses (L1, L2, and L3), beamsplitter (BS), aperture (A), spectrometer (S), and optical fiber patch cord (F) (b) Schematic view of the brain showing light interacting with the target tissue. The light is coupled from the optical fiber patch cord ferrule to the implant ferrule connected using a ceramic sleeve. The IR LEDs are powered using a synchronization (sync) pulse. A camera is used to record the behavior of the animal and the sync pulses. (c) The simulation model represents the tissue block, the fiber whose far end is angled at 16 deg, and the photon trajectory during light tissue interactions. Zoomed-in insets shows the core, cladding, source-detector geometry, and the 16 deg fiber far end.

The animal experiments carried out were in accordance with the guidelines of the Canadian Council on Animal Care and were approved by the Institutional Animal Care and Use Committee of the University of Calgary. The optical setup of SFS has lenses - L1, L2, and L3, an aperture (A), and a beamsplitter (BS), as shown in Fig. 1(a).^6^ The collected light is detected using a spectrometer (S, MayaPro 2000, Ocean Optics). A microcontroller was used to synchronize SFS with behavioral recording cameras and FS generator through infrared LED pulses (peak wavelength of 940 nm) recorded by the spectrometer.

Six Ai-148 mice were implanted with custom-made 16-deg angle polished ferrules (Doric Lenses, Canada) at the PVN region of the brain through a stereotactic procedure.^6^^,^^20^^,^^22^ Based on our previous study, the 16-deg angle was determined to reduce the back reflection during the coupling of light into the fiber while minimizing the tip sharpness that can cause brain damage.^23^ During the surgery, mice are first anesthetized and maintained under anesthesia using an isoflurane-oxygen mixture. The region between the ears is shaved and disinfected using 70% alcohol and Betadine. A hole is made in the skull to target the PVN region (anterior–posterior (AP) = −0.7 mm, dorsal–ventral (DV) = −4.7 mm from the dura and medial-lateral (ML) = −0.2 mm from the bregma), following which the implant ferrule is lowered.^20^^,^^22^ Dental cement and meta bond are used to secure the ferrules on the skull of the animal. After post-operative care, animals were then used for recordings. During our experiments, animals were 6 to 8 weeks old at the time of surgery and 12 to 16 weeks old at the time of recordings. The recordings were performed using optical fiber patch cords of 400  μm core, 0.53 NA, and 2 m in length with 16-deg angle polished ends (Doric Lenses, Canada). A cross section of the brain where light is interacting with the tissue is shown in Fig. 1(b). Prior to recording in all experiments, the animals were left to habituate in the recording room for at least 20 min. First, spectra were measured using the spectrometer with the fiber in air ($I_{a}^{M}$) and in glycerine ($I_{g}^{M}$). Then the optical fiber patch cord was connected to the implant ferrule using a ceramic sleeve, and spectra were measured from the brain ($I_{b}^{M}$). $I_{b}^{M}$ refers to the backscatter from the tissue. Before connecting the patch cord to the implant ferrule, a drop of glycerine is used as an index-matching medium to maximize light coupling into the implant ferrule. During recording spectra, we set the typical exposure times (e.g., 20 to 25 ms) and number of spectra averaged of the spectrometer (e.g., 4 to 5) to maintain a sampling rate of about 10 Hz. After recording from an animal, the fiber tip is cleaned using water. $I_{a}^{M}$ and $I_{g}^{M}$ are then remeasured before recording $I_{b}^{M}$ from next animal. The measured reflectance spectrum ($R_{\mathrm{SF}}^{M}$) is then calculated as

$$R_{\mathrm{SF}}^{M}=\frac{I_{b}^{M}-I_{g}^{M}}{I_{a}^{M}-I_{g}^{M}}.$$ (1)

Similar to previous work from our group, the $R_{\mathrm{SF}}^{M}$ spectra were fit to an empirical model of photon transport in tissue to obtain % ${\mathrm{sO}}_{2}$.^6^^,^^17^^,^^19^^,^^24^^–^^26^ Briefly, the method involves fitting the $R_{\mathrm{SF}}^{M}$ using a non-linear least square fitting approach in MATLAB to extract % ${\mathrm{sO}}_{2}$, which is one of the parameters of the model. During the $R_{\mathrm{SF}}^{M}$ calculation, we select $I_{a}^{M}$ and $I_{g}^{M}$ which results in a lower fitting error. All scripts used for data processing have been provided in the online repository mentioned in Sec. 7.

### 2.1 Experiment-1

Certain experiments in neuroscience involve transitioning animals from one arena to another, where large motion artifacts can be possible due to the transfer process between arenas.^20^ We recorded hemodynamics data while we transitioned animals from arena-1 after 5 min to arena-2 for 5 min and back to arena-1 for a duration of 5 min. Three animals were used for this experiment.

### 2.2 Experiment-2

Footshocks (FS) are applied to mice in experiments where stress-based behavior is studied or in fear conditioning studies.^21^ Earlier studies have shown robust responses recorded during fluorescence photometry from the periventricular nucleus (PVN region) of the hypothalamus.^20^^,^^27^ During an FS, the mouse jumps aggressively, which causes movement of the optical fiber patch cord (F), which creates a situation where large effect of motion artifact can be speculated. FS were generated using ANY-Maze system and were synchronized to the SFS recording using the IR LED [shown in Fig. 1(a)]. Ten FS of 2-s long duration were delivered to the animal in the foot-shock cage with a 30-s inter-stimulus period. An FS trial was defined as 8 s prior to the onset of FS to 22 s post onset of FS. This resulted in a total trial duration of 30 s. Three animals were used for this experiment.

### 2.3 Monte Carlo Simulation

To mimic light-tissue interaction in the SFS, we developed a simulation model in the non-sequential simulation mode of Zemax OpticStudio. In the simulation model, we omitted certain elements that remain static and do not interfere with the signals we are concerned with in these simulations. A rectangular volume was used to simulate the brain tissue. An optical geometry consisting of a source and detector was set up to couple and detect light respectively from the brain tissue [as shown in Fig. 1(c)]. The far end of the optical fiber had a 16 deg end face and was embedded in the tissue to mimic the experimental setting closely (See Sec. 2).^23^ A light source was configured to launch $10^{6}$ photons into the tissue through the optical fiber. A justification for the number of photons is included in the supplementary data (Fig. S1 in the Supplementary Material). The refractive index of the brain tissue and optical fiber was modelled using Cauchy’s formula using data from the literature.^28^^,^^29^ Tissue absorption of the brain tissue was modeled using the equation $\mu_{a}=\mathrm{BV}({\mathrm{sO}}_{2}\mu_{\mathrm{HbO}}+(1-{\mathrm{sO}}_{2})\mu_{\mathrm{Hb}})$, where $\mu_{\mathrm{HbO}}$, $\mu_{\mathrm{Hb}}$ are the absorption coefficients of oxyhemoglobin and deoxyhemoglobin, and BV is the blood volume fraction.^10^ Scattering was modelled using an intralipid scattering model with the Henyey–Greenstein (HG) scattering phase function.^30^^,^^31^ The HG function is given by $p(\theta )=\frac{1}{4\pi }(\frac{1-g^{2}}{(1+g^{2}-2g\;\cos(\theta ){)}^{3/2}})$ where p(θ) represents the probability of scattering in the direction of the scattering angle (θ) and g is the anisotropy factor.^30^ We modelled the anisotropy factor (g) of intralipid using the equation $g(\lambda )=1.1-(0.58\times 10^{-3})\times \lambda$ leading to values between ∼0.7−0.87 for the wavelength range ∼400−700  nm.^31^ Using these values in the HG phase function reveals forward scattering characteristics, which agree with the scattering properties of biological tissues.^32^ The simulation model, along with a sample photon trajectory, is shown in Fig. 1(c). During simulations, the % ${\mathrm{sO}}_{2}$ and BV were varied to calculate different absorption coefficients. The absorption coefficient and the scattering coefficients were used to calculate the mean free path (MFP) and transmission parameter, which along with g are inputs to the Zemax OpticStudio software. Changes in optical transmission during motion artifact simulations were modeled by forcing a transmission coating at the fiber/brain interface. A macro written in Zemax programming language was used to automate the simulation process.

#### 2.3.1 Static Monte Carlo simulations

Ten MC simulations were run for constant tissue properties (35% ${\mathrm{sO}}_{2}$, and ρ=1%) for various wavelengths (specified in Secs. 3.1 and 3.3).^6^^,^^33^ Simulations were initially run to model the light collected with the fiber in air ($I_{a}^{S}$) and glycerine ($I_{g}^{S}$) followed by in tissue ($I_{b}^{S}$). Data generated in Zemax Opticstudio were processed in MATLAB to yield simulated $R_{\mathrm{SF}}^{S}$ using the following equation:

$$R_{\mathrm{SF}}^{S}=\frac{I_{b}^{S}-I_{g}^{S}}{I_{a}^{S}-I_{g}^{S}}.$$ (2)

The calculated $R_{\mathrm{SF}}^{S}$ was used to calculate % ${\mathrm{sO}}_{2}$ using the empirical model as described in Sec. 2. Prior to fitting, we additionally interpolated the simulated data in MATLAB to improve the fitting process.^25^

#### 2.3.2 Dynamic Monte Carlo simulations

During dynamic hemodynamic simulation, we modelled blood absorption changes across time at two different wavelengths (i.e., 680 and 545 nm). The 545 nm wavelength was chosen to evaluate changes due to hemodynamics due to its relatively large sensitivity to hemoglobin absorption. The 680 nm wavelength was used to observe effects due to non-hemodynamic changes such as motion artifacts due to its lower sensitivity to hemoglobin absorption. We performed simulation with and without including motion artifacts. Hemodynamic signals have been previously modeled using hemodynamic response functions (HRFs).^34^ We used definitions provided by Kamran et al. to generate the dynamics of the temporal variation of the signal.^35^ The hemodynamic response function [HRF(t), Fig. S2(a) in the Supplementary Material] was produced by convolving a canonical hemodynamic impulse response function [h(t)] with the stimulus function [s(t), Fig. S2(a) in the Supplementary Material], i.e., HRF(t)=h(t)*s(t). The canonical hemodynamic response function is described as

$$h(t)=[\frac{t^{\alpha_{1}-1}\beta_{1}^{\alpha_{1}}e^{-\beta_{1}t}}{\Gamma (\alpha_{1})}-\frac{t^{\alpha_{2}-1}\beta_{2}^{\alpha_{2}}e^{-\beta_{2}t}}{6\Gamma (\alpha_{2})}],$$ (3)

where t represents the time points; $\beta_{1},\beta_{2}$, $\alpha_{1},\alpha_{2}$ are parameters; and Γ() refers to the gamma function. Next, the final physiological hemodynamic response (R(t)) is written as a weighted superposition of the HRF(t), sinusoidal terms and a DC bias ($p_{o}$) as described in Eq. (4). The sinusoidal terms in Eq. (4) are representative of cardiac pulsations, respiration, and Mayer waves with frequencies $f_{c}$, $f_{r}$, and $f_{m}$. We used $f_{c}=1\;\mathrm{Hz}$, $f_{r}=0.2\;\mathrm{Hz}$, and $f_{m}=0.07\;\mathrm{Hz}$ for our simulations [Fig. S2(b) in the Supplementary Material].^35^ The terms $p_{c}$, $p_{r}$ and $p_{m}$, and $p_{1}$ are weighting factors. In our work, we have used R(t) to model the changes in light intensity recorded by the SFS (i.e., $I_{b}^{M}$)

$$R(t)=p_{o}+p_{1}\mathrm{HRF}(t)+p_{c}\;\sin(2\pi f_{c}t)+p_{r}\;\sin(2\pi f_{r}t)+p_{m}\;\sin(2\pi f_{m}t).$$ (4)

The normalized R(t), represented as y(t) [shown in Fig. S2(c) in the Supplementary Material], is appropriately scaled to simulate changes in the concentration of oxy ($\Delta C_{\mathrm{HbO}}$) and deoxyhemoglobin ($\Delta C_{\mathrm{Hb}}$), as shown in Fig. S2(d) in the Supplementary Material. Following this, the absorption coefficient of tissue is calculated as

$$\mu_{a}(t)=\mu_{\mathrm{HbR}}(\Delta C_{\mathrm{HbR}}\cdot y(t)+(C_{\mathrm{HbT}}/2))+(\Delta C_{\mathrm{HbO}}\cdot y(t)+(C_{\mathrm{HbT}}/2))\mu_{\mathrm{HbO}},$$ (5)

where $C_{\mathrm{HbT}}$ is the concentration of total hemoglobin. We used $C_{\mathrm{HbT}}=50\;\mu M$, and $\Delta C_{\mathrm{HbO}}=10\;\mu M$ and $\Delta C_{\mathrm{HbR}}=2.5\;\mu M$ for the simulations. These concentrations are in agreement with experimentally observed values.^36^^,^^37^ $I_{b}^{S}$ at 545 nm and 680 nm was calculated using MC simulations at a time resolution of 10 Hz with a stimulus (s(t)) from 2 to 4 s. To simulate motion artifacts, a second series of simulations were done with a 96% transmission coating that was applied on the fiber/brain interface in the model between 2 and 3.5 s.

### 2.4 Quantifying Changes in Signals

#### 2.4.1 Quantifying shifts in reflectance spectra ($R_{\mathrm{SF}}$) between experimental data and simulation

We quantified the offset-like changes observed in the $R_{\mathrm{SF}}$ using root mean square deviation (RMSD) given by the following equation:

$${\mathrm{RMSD}}_{T}=\sqrt{\frac{1}{N}{\left(\overset{N}{\underset{i=1}{\sum }}(R_{\mathrm{SF},T}(\lambda_{i})-R_{\mathrm{SF},\mathrm{ref}}(\lambda_{i}))\right)}^{2}},$$ (6)

where ${\mathrm{RMSD}}_{T}$ is the RMSD calculated for a test curve ($R_{\mathrm{SF},T}$) with respect to a reference curve ($R_{\mathrm{SF},\mathrm{ref}}$), and N is the number of points in $R_{\mathrm{SF}}$.

#### 2.4.2 Dissimilarity metric

To quantify the magnitude of changes seen in $I_{b}^{M}$ due to motion artifacts, we developed a DM^25^ defined by the following equation:

$$\mathrm{DM}(i)=\frac{\overset{\lambda_{q}}{\underset{\lambda =\lambda_{p}}{\sum }}I_{b}^{M}(\lambda ,i)-\frac{1}{N}\overset{i+\frac{N}{2}}{\underset{j=i-\frac{N}{2}}{\sum }}\overset{\lambda_{q}}{\underset{\lambda =\lambda_{p}}{\sum }}I_{b}^{M}(\lambda ,j)}{\frac{1}{N}\overset{i+\frac{N}{2}}{\underset{j=i-\frac{N}{2}}{\sum }}\overset{\lambda_{q}}{\underset{\lambda =\lambda_{p}}{\sum }}I_{b}^{M}(\lambda ,j)}.$$ (7)

DM represents the normalized instantaneous difference in the intensity of collected light ($I_{b}^{M}$) in a wavelength range from $\lambda_{p}$ to $\lambda_{q}$ from the average intensity over a window of length N centered around a sample i. We set $\lambda_{p}=670\;\mathrm{nm}$ and $\lambda_{q}=680\;\mathrm{nm}$ which represents the hemodynamic insensitive region (Sec. 3.1). We then calculate the standard deviation of DM ($\sigma_{\mathrm{DM}}$) to evaluate the effect of motion artifacts. A higher $\sigma_{\mathrm{DM}}$ corresponds to a higher variation in the wavelength insensitive region of $I_{b}^{M}$, indicating a higher contribution from motion artifacts.

#### 2.4.3 Change in backscatter intensity ($I_{b}^{M}$)

545 nm wavelength is an isosbestic wavelength of hemoglobin with a relatively large sensitivity to hemoglobin due to the large absorption coefficient of hemoglobin. We refer to the measured intensity of light backscattered from the brain as the backscatter intensity ($I_{b}^{M}$). We calculated the % change in $I_{b}^{M}$ at 545 nm relative to the mean of the baseline period as an indicator of blood perfusion for analyzing both experimental and simulation data.^19^ For experiment 2 (Sec. 2.1), the baseline period was defined as the 10 s prior to the onset of FS, while for the dynamic simulations (Sec. 2.3.2), it was defined as the 2 s period prior to stimulus onset.

## 3 Results

We first identify a wavelength region that is relatively independent of hemodynamics (Sec. 3.1) and then quantify the relative magnitude of motion artifacts (Sec. 3.2). We next simulate motion artifacts using MC simulations to understand experimental findings (Sec. 3.3). Next, a model for light propagation through SFS and the tissue is proposed and extended to a MAC algorithm (Sec. 3.4). The MAC is first validated using simulation data (Sec. 3.5), then its application is illustrated in experimental data (Sec. 3.6).

### 3.1 Identifying a Hemodynamics-Independent Wavelength Region

**Fig. 2:**
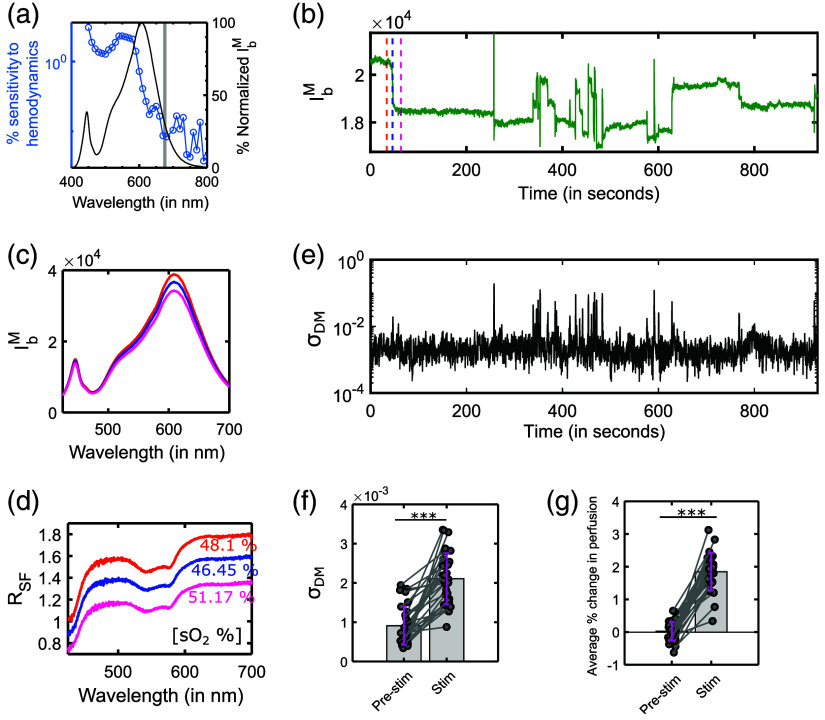
(a) Percent (%) sensitivity of collected light to tissue absorption during simulations and % normalized intensity of collected backscattered light ($I_{b}^{M}$) by the SFS during the experiment. The grey-shaded region represents 670 to 680 nm. (b) Changes in $I_{b}^{M}$ at 545 nm across time for experiment-1 for an animal. (c) $I_{b}^{M}$ for three times points marked with different colours (pink, blue, and orange). (d) Corresponding $R_{\mathrm{SF}}^{M}$ and % ${\mathrm{sO}}_{2}$ for the three time points (e) Standard deviation of DM ($\sigma_{\mathrm{DM}}$) calculated using data from (b). (f) $\sigma_{\mathrm{DM}}$ for experiment-2 between the pre-stim and stim conditions. (3 mice, 10 trials per mouse) (g) Peak changes in perfusion for both pre-stim and stim conditions (3 mice, 10 trials per mouse). *** represents p<0.0001.

The spectra of light collected through SFS are dependent on the incident spectra, tissue properties (such as scattering and absorption), and motion artifacts. Hemodynamic changes are strongly affected by blood absorption changes, which are wavelength-dependent. Motion artifacts can cause wavelength-independent changes in contrast to wavelength-specific changes during hemodynamics.^6^^,^^25^^,^^36^ To quantify motion artifacts, the objective in this section was to identify a wavelength region where we collected a reasonable amount of light that was minimally sensitive to blood absorption changes. We performed 10 static MC simulation runs for two cases—(i) 1% BV, 35% ${\mathrm{sO}}_{2}$ with intralipid scattering model, and (ii) only intralipid scattering model (i.e., $\mu_{a}=0$) using the simulation model described in Sec. 2.3.1 for 55 wavelengths between 450 and 790 nm. We used $10^{6}$ photons for the MC simulations to minimize the inherent MC simulation noise [Fig. S1(a) in the Supplementary Material]. We averaged the 10 simulation runs to further reduce the noise. Figure 2(a) shows each wavelength’s sensitivity to hemodynamics, defined as the absolute % change in the light collected between the scattering with absorption and the scattering-only cases. The figure also shows the normalized spectrum of the light incident on tissue with our experimental setup. Our simulations showed that while wavelengths beyond 700 nm showed the least sensitivity to changes in blood absorption, we collect very little light beyond 700 nm [Fig. 2(a)]. As a tradeoff between the quality of the signal and its independence from hemodynamics, we chose the 670 to 680 nm region as largely representing the motion artifacts.

### 3.2 Motion Artifacts in the Measured Backscatter Intensity ($I_{b}^{M}$) Signals

In this section, we analyze the $I_{b}^{M}$ signals acquired during experiment-1. Figure 2(b) shows the $I_{b}^{M}$ signals at 545 nm for a single animal during experiment-1. We observe that the $I_{b}^{M}$ shows large, abrupt changes at multiple time points. To investigate these changes further, Figs. 2(c) and 2(d) show $I_{b}^{M}$ and $R_{\mathrm{SF}}$ across the entire spectrum for the three-time points marked in Fig. 2(b) respectively. We observed wavelength-wide, offset-like changes in $R_{\mathrm{SF}}$ during the abrupt changes. Since hemodynamics is a slow process and causes wavelength-specific changes, the rapid broadband changes observed in Figs. 2(b)–2(d) are likely artifactual. We quantified the offset-like changes observed in Fig. 2(d) using RMSD as described in Sec. 2.4.

To calculate RMSD, wavelengths ranging from ∼448−712  nm, with 1 nm spacing were used, giving a total N=256 points. The RMSD of $R_{\mathrm{SF}}$ shown in blue and pink with respect to the one in orange is 0.19 and 0.41, respectively. Figure 2(d) shows the corresponding % ${\mathrm{sO}}_{2}$ extracted for each of the $R_{\mathrm{SF}}$ using the empirical model as described in Sec. 2. Given that the % ${\mathrm{sO}}_{2}$ depends on the spectrum’s shape, the % ${\mathrm{sO}}_{2}$ extracted is expected to be relatively immune to motion artifacts.^38^ The immunity of % ${\mathrm{sO}}_{2}$ to transmission changes has been further addressed in Sec. 3.3. Therefore, in this work, we focus on understanding and mitigating motion artifacts in the perfusion signal.

#### 3.2.1 Using dissimilarity metric to quantify motion artifacts

To illustrate the utility of DM (described in Sec. 2.4.2) in quantifying motion artifacts, we partitioned the data using a sliding window into 1-s chunks, which were then used to calculate DM values. The standard deviation of DM ($\sigma_{\mathrm{DM}}$) over those chunks is plotted in Fig. 2(e). It is observed that $\sigma_{\mathrm{DM}}$ peaks when there is an abrupt change in the signal as shown in Fig. 2(b). This indicates that $\sigma_{\mathrm{DM}}$ can be used as a reliable measure of changes in the signals.

Next, we used DM to evaluate the extent of motion artifact during experiment-2. FS in freely moving mice induce short episodes of panic and elicit jumping behavior. The thirty FS trials (3 mice, 10 trials per mouse) were divided into two data groups. The pre-stim condition consisted of data for the first 8 s of the trial, where we expect minimal motion artifact. The stim condition consisted of data 8 s post onset of the FS where large motion artifacts are possible.

DM was calculated for the two conditions followed by the calculation of $\sigma_{\mathrm{DM}}$. Figure 2(f) shows the $\sigma_{\mathrm{DM}}$ for both conditions displayed in a pair-wise plot. We observed that there was a statistically significant difference (p<0.0001, using Wilcoxon signed rank test) in the amount of motion artifact between the two conditions with larger artifacts after the onset of the FS (i.e., for the stim condition). FS in mice have been shown to increase blood flow in the PVN due to an increase in the activity of PVN neurons of the hypothalamus (unpublished data^20^^,^^22^). We calculated the % change in backscatter intensity based on Sec. 2.4.3. This was used to calculate the average % backscatter change in 300 ms windows prior to the start of and just before the end of FS to represent the pre-stim and stim periods, respectively. Since an increase in perfusion leads to a decrease in backscatter intensity, Fig. 2(g) illustrates the negative of % change in backscatter intensity. We observed an increase in the perfusion response (p<0.0001, using Wilcoxon signed rank test) in the stim condition compared to pre-stim. Since motion artifacts impact $I_{b}^{M}$ [Figs. 2(b)–2(d)], the increase observed in the perfusion in the stim condition can be considered a superposition of effects due to true hemodynamics and motion artifacts.

### 3.3 Simulating Motion Artifacts Using Monte Carlo Simulations

From Sec. 3.2, we observed that motion artifacts caused changes in $I_{b}^{M}$. As described in Sec. 3.2, such motion-induced changes in optical signals can be modelled as changes in the transmission of the light path.^25^ Changes in transmission can occur at optical interfaces in the lightpath that are susceptible to motion, e.g., the fiber implant ferrule interface, fiber-brain interface, or movement of the optical fiber. In this section, we present MC simulations with transient changes in the lightpath transmission to model variations in $I_{b}^{M}$, $R_{\mathrm{SF}}^{M}$, and % ${\mathrm{sO}}_{2}$ that can occur during motion artifacts. The simulated tissue back-scatter is represented as $I_{b}^{S}$, in contrast to the measured tissue backscatter that is referred to as $I_{b}^{M}$. With $10^{6}$ photons in the simulation, Fig. S1(b) in the Supplementary Material shows that the hemodynamic features (e.g., hemoglobin absorption valleys at ∼550  nm and ∼570  nm) in the simulated $I_{b}^{S}$ were distinctly visible. This shows that the simulated hemodynamic features were well over the noise due to inherent randomness in MC simulations which is important for % ${\mathrm{sO}}_{2}$ estimation.

**Fig. 3:**
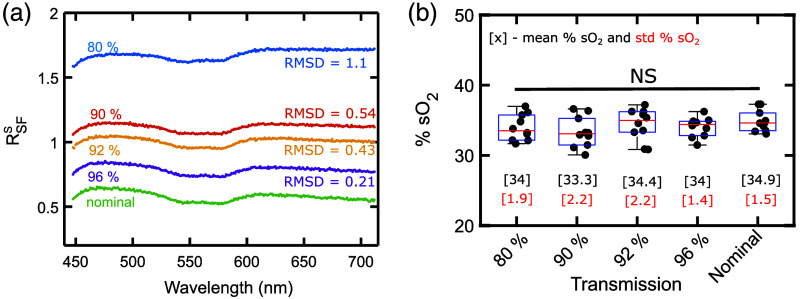
(a) Various simulated $R_{\mathrm{SF}}^{S}$ for different transmission changes (i.e., 80%, 90%, 92%, 96%, and nominal), and the corresponding RMSD calculation. (b) Boxplot showing the median % ${\mathrm{sO}}_{2}$ value (red line) for 10 MC simulations each for different transmissions. The height of the box represents the IQR, which is a measure of the spread of the data. The raw data (points), mean and standard deviation (std) across the simulations have been also depicted.

The tissue oxygenation and blood volume were set to 35 % ${\mathrm{sO}}_{2}$ and 1 % BV, respectively, while other simulation properties were the same as described in Sec. 2.3,^33^ While the transmission can change anywhere along the lightpath, we simulated changes by adding a transmission coating at the fiber-tissue interface. To stay consistent with how $R_{\mathrm{SF}}$ is calculated, we first simulated the nominal back-reflection in glycerine ($I_{g}^{S}$) and air ($I_{a}^{S}$). Next, MC simulations to extract the simulated tissue backscatter were carried out at coating transmission values 96%, 92%, 90%, and 80%. The nominal case representing no artifacts was simulated without any coating. Resulting $R_{\mathrm{SF}}^{S}$ based on calculations described in Sec. 2.3.1 are shown in Fig. 3(a). To quantify the relative shift in the spectrum, we used the RMSD calculation described in Sec. 2.4.1 using $R_{\mathrm{SF}}^{S}$ for nominal transmission as the reference.

Figure 3(b) shows the boxplot for the % ${\mathrm{sO}}_{2}$ calculated for various transmissions based on methods described in Sec. 2.3.1. The height of the boxplot is representative of the interquartile range (IQR), which is a measure of the spread of the data. As the fitting process is sensitive to noise, each simulation was repeated 10 times resulting in 10 values of % ${\mathrm{sO}}_{2}$ for each transmission. The small variations in the IQR can be attributed to the fitting noise and inherent variability during MC simulations [Fig. S1(b) in the Supplementary Material]. To test if the changes in the transmission affect the % ${\mathrm{sO}}_{2}$ among all the different transmission levels, we performed non-parametric one-way analysis of variance (ANOVA) using the Kruskal–Wallis test. We observed a p-value of 0.44 implying that the % ${\mathrm{sO}}_{2}$ for various transmissions was not statistically significantly different from each other.

The offset-like shifts observed in the simulated reflectance spectra $R_{\mathrm{SF}}^{S}$ shown in Fig. 3(a) during transmission changes were also observed in the measured reflectance spectra $R_{\mathrm{SF}}^{M}$ shown in Fig. 2(d). While there are certain differences in the characteristic hemodynamic features (e.g., shape of the curves and slope), the focus here is to characterize large non-hemodynamic offset like changes. Therefore, to assess the offset-like shifts with respect to the nominal transmission $R_{\mathrm{SF}}^{S}$ quantitatively, we calculated RMSD [shown in Fig. 3(a)]. The RMSD observed in the experiment (i.e., 0.19 and 0.41, described in Sec. 3.2) is comparable to what we observed in Fig. 3(a) (i.e., 0.21 to 1.1). This provides evidence to support the idea that transmission changes in the lightpath could be used to model offset-like changes during motion artifacts.

### 3.4 Motion Artifact Correction Algorithm for Reflectance Signals

In this section, we propose an MAC algorithm to correct changes in the perfusion signal due to motion artifacts caused by transmission changes. Equation (8) describes the intensity of light measured by SFS from the brain ($I_{b}^{M}$) during recording that accounts for Fresnel reflection at the optical interfaces and tissue backscatter. A detailed explanation of the model is provided elsewhere^25^

IbM=I·(RaNf)+I·(TaNf)·T·(RfFb)·T·(TfNa)+RSF·Ib·(TbFf)·T·(TfNa). (8)

In Eq. (8), the incident spectra just before coupling to the fiber is denoted by I. Rxzy and Txzy refer to the transmission and reflection coefficients, respectively, as light goes from medium x to y at location z. The media is indicated by superscripts: a, f, b refer to air, fiber, and brain. The locations are indicated by subscripts: N and F refer to the near end (where light enters the fiber) and the far end (where light enters the brain) of the fiber, respectively. T is the fiber transmission. $R_{\mathrm{SF}}$ is the actual reflectance spectrum of the tissue. Further, Ib=I·(TaNf)·T·(TfFb) is the light intensity entering the brain at the far end.

We showed in Sec. 3.1 that $I_{b}^{M}$ at 670-680 nm wavelength has a little contribution from hemodynamics and therefore largely depends on changes due to Fresnel reflection. This implies that, the RSF·Ib·(TbFf)·T·(TfNa) term in Eq. (8) contributes negligibly as $R_{\mathrm{SF}}\approx 0$. Further, in Sec. 3.2 we have uncovered that changes observed in the 670 to 680 nm can be used as a proxy for motion artifacts. To simplify calculations, we use 680 nm to represent the range. Therefore, the light collected at 680 nm becomes

Ib at 680  nmM=I·(RaNf)+I·(TaNf)·T·(RfFb)·T·(TfNa). (9)

$I_{b\;\text{at}\;680\;\mathrm{nm}}^{M}$ can be used to approximate all transmission-based changes (i.e., motion artifacts) in the recorded artifactual backscatter signal ($I_{b}^{M}$). Therefore, using Eqs. (8) and (9), we can write $I_{b}^{M}=m\times I_{b\;\text{at}\;680\;\mathrm{nm}}^{M}+I_{b\;\text{true}}^{M}$, where m is a wavelength dependent factor and IbtrueM=RSF·Ib·(TbFf)·T·(TfNa) is the back-scatter signal of interest. The parameter m is important to scale wavelength-dependent changes observed in the $I_{b\;\text{at}\;680\;\mathrm{nm}}^{M}$ wavelength to the $I_{b}^{M}$ at other wavelengths. We calculate the corrected $I_{b}^{M}$ (i.e., $I_{b\;c}^{M}$) as IbcM=IbtrueM=IbM−m×n^(t) where n^(t)=Ib at 680  nmM−⟨Ib at 680  nmM⟩ and $\left\langle I_{b\;\text{at}\;680\;\mathrm{nm}}^{M}\right\rangle$ represents the mean of the time series. The parameter m is extracted using a linear regression-based approach.^25^

### 3.5 Motion Artifact Correction Validation through Simulation Data

To further validate the MAC algorithm we simulated the changes in the tissue backscatter intensity ($I_{b}^{S}$) measured by SFS at two wavelengths, i.e., 545 and 680 nm as a function of tissue absorption as described in Sec 2.3.2.

**Fig. 4:**
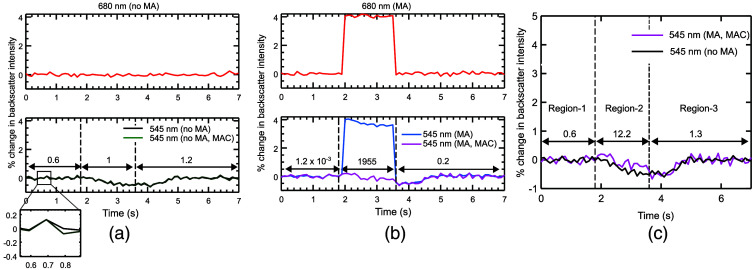
Simulations showing % changes in backscatter intensity (a) at 680 nm, 545 nm without motion artifact (MA) and corresponding corrected 545 nm data using MAC algorithm. (b) shows data at 680 and 545 nm during motion artifact, and corresponding corrected data at 545 nm using the MAC algorithm. (c) A comparison of 545 nm data processed by MAC algorithm and 545 nm without motion artifact. Regions 1, 2, and 3 are identical in all figures, as indicated in panel (c). MSE (normalized by $10^{-5}$, i.e., 0.6 represents $0.6\times 10^{-5}$) between the traces in each region is indicated in the plots.

$I_{b}^{S}$ at 545 nm represents the perfusion signal, and that at 680 nm represents the correction signal. Figure S1(c) in the Supplementary Material shows that the $10^{6}$ photons used for the simulation were sufficient to produce a change in the $I_{b}^{S}$ at 545 nm which was well over the noise due to inherent randomness during MC simulations. There was no significant change seen in $I_{b}^{S}$ at 680 nm due to its insensitivity to hemodynamics as per the proposed MC simulation model (Sec. 2.3). For Fig. 4, the $I_{b}^{S}$ was processed to calculate the % change in backscatter intensity with respect to ∼2  s baseline period prior to stimulus-evoked change (Sec. 2.4.3). Figure 4(a) shows the results from the simulation without motion artifacts (MA). As expected, the 680 nm, signal has no features and correcting the perfusion signal does not change it. Figure 4(b) shows the case with artifacts. Large variations can be seen in both channels and using the correction described in Sec. 3.4 recovers the perfusion measurement. Figure 4(c) compares the ideal trace with the corrected artifactual trace.

We performed mean squared error (MSE) calculations to evaluate the effectiveness of the MAC algorithm. We calculated the MSE between the traces shown in the bottom panel of Figs. 4(a)–4(c). This was done by segregating the plots to three regions – (i) region-1 (t=0 to 1.8 s, prior to motion artifact), (ii) region-2 (t=1.8 to 3.6 s, during motion artifact) and (iii) region-3 (t=3.6 to 7 s, after motion artifacts) as shown in Fig. 4(c). We observed a small MSE for the region-1 and region-3, as shown in Figs. 4(a)–4(c), indicating that the correction does not significantly change signal which is free of artifacts. During motion artifact [region-2, Fig. 4(b)], the MSE increased by a factor of about 4 to 6 orders of magnitude in comparison to the MSE values in regions-1 and 2, highlighting the large effect of motion. The MSE was reduced by about 2 orders of magnitude after MAC for region 2 in Fig. 4(c).

### 3.6 Applications in Experimental Data

**Fig. 5:**
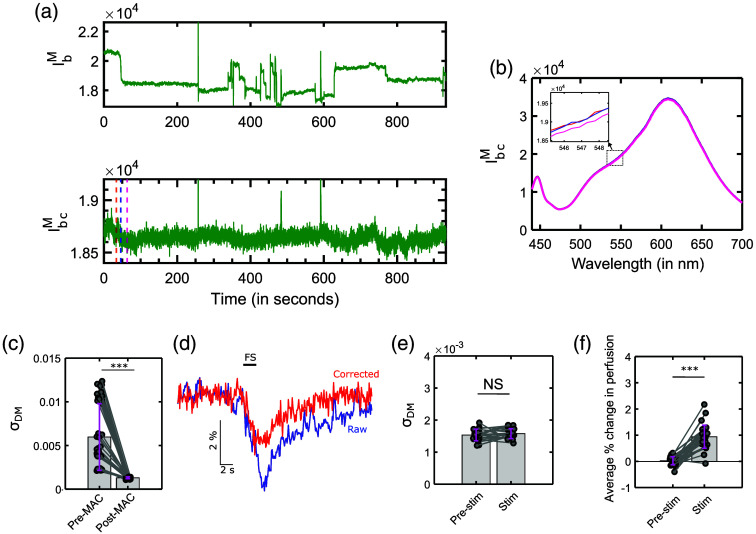
(a) Raw ($I_{b}^{M}$) and corrected ($I_{b\;c}^{M}$) backscatter intensity at 545 nm, and the three-time points identical to Fig. 2. (b) The corresponding $I_{b\;c}^{M}$ for three-time points after correction in contrast to Fig. 2(c). (c) Cumulative $\sigma_{\mathrm{DM}}$ for the whole time series in the experiment - 1 (3 mice, $15\;\sigma_{\mathrm{DM}}$ values per mouse). (d) The corrected and original traces for % change in backscatter intensity for single trial. (e) $\sigma_{\mathrm{DM}}$ for experiment-2 post-MAC for the pre-stim and stim condition (3 mice, 10 trials per mouse) (f) The average % change in perfusion for the two conditions pre-stim and stim (3 mice, 10 trials per mouse). *** represents p<0.0001.

We first show the application of MAC for data from experiment 1, followed by experiment 2. Figure 5(a) shows the raw ($I_{b}^{M}$) and corrected ($I_{b\;c}^{M}$) backscatter intensity at 545 nm. As described earlier in Sec. 3.2, we observed that motion artifacts cause abrupt changes in the $I_{b}^{M}$ as shown in Fig. 5(a) that are minimized following correction in $I_{b\;c}^{M}$.

Figure 5(b) shows the spectrum of the collected light post-correction (i.e., $I_{b\;c}^{M}$) where the shifts observed in the spectrum (i.e., $I_{b}^{M}$) in Fig. 2(c) prior to correction have been minimized.

Further, the $I_{b}^{M}$ and $I_{b\;c}^{M}$ time series were broken down to 15 60-s-long chunks for each animal. The 45 chunks were used to compute a series of DM values. Figure 5(c) shows the $\sigma_{\mathrm{DM}}$ calculations prior to correction, i.e., pre-MAC, and after correction, i.e., post-MAC. We observed that the post-MAC $\sigma_{\mathrm{DM}}$ was lower than the pre-MAC $\sigma_{\mathrm{DM}}$. A Wilcoxon signed rank was used to compare the pre-MAC vs post-MAC data, which revealed a statistically significant difference exists between the two (p<0.0001). This shows that the MAC is successfully able to minimize the variations present in the backscatter signal.

Next, we applied MAC for experiment-2. Figure 5(d) shows % change in backscatter intensity calculated relative to the 10 s baseline period for a single FS trial. We observe that the peak % change in perfusion is less for the corrected trace (in red) than the raw trace (in blue). This decrease observed during correction can be attributed to the combination of (i) a decrease in motion artifacts due to the correction, and (ii) attenuation in the perfusion signal due to correction using 680 nm wavelength, which is not completely insensitive to absorption due to hemodynamics, i.e., due to the non-zero absorption coefficient of hemoglobin at 680 nm.

Identical to the approach in Fig. 2(f), in Fig. 5(e) we used the $I_{b\;c}^{M}$ to calculate $\sigma_{\mathrm{DM}}$ for pre-stim and stim data. We observed that both conditions led to comparable $\sigma_{\mathrm{DM}}$. A comparison using a paired Wilcoxon signed rank test showed that the difference is not statistically significant (p=0.2). Figure 5(f) shows the % change in perfusion between pre-stim and stim conditions calculated after correction using $I_{b\;c}^{M}$ similar to the comparison of the raw data in Fig. 2(g). Post correction plot in Fig. 5(f) shows that the two are still significantly different (p<0.0001, using Wilcoxon signed-rank test), indicating that the correction does not obscure the effect of stimulation.

## 4 Discussion

An understanding of motion artifacts is critical to discriminate between signal and noise. Motion artifact mitigation is an active area of research across the biomedical sciences, including light-based techniques. However, knowledge of motion artifacts and how they impact signals during fiber-based measurements, especially SFS is limited and is the focus of our work. The large rapid changes observed during our experiments [e.g., in Figs. 2(b)–2(d)] have been observed by researchers during optical probe-based diffuse optical imaging, and fiber-based NIRS, and have been recognized as motion artifacts.^11^^,^^12^^,^^14^^,^^15^

There can be several reasons for the shifts observed in the collected light ($I_{b}^{M}$) and the calculated reflectance ($R_{\mathrm{SF}}^{M}$) such as those shown in Figs. 2(c) and 2(d). First, movement at the fiber patch cord ferrule/implant ferrule or the fiber/brain interface can lead to changes in the optical coupling at the interface. This will change both the light exiting and entering the fiber, leading to artifacts. Changes in pressure on the tissue due to motion can cause alterations to local blood flow leading to changes in $I_{b}^{M}$ and $R_{\mathrm{SF}}$.^16^^,^^39^ Further, movement of the fiber has been known to induce artifacts which can be an additional factor causing changes in light transmission and collection.^13^

The first objective of this work was to discriminate between true hemodynamics signals and motion artifacts. This was achieved by finding a wavelength region in the collected light, which is relatively independent of tissue backscatter and more sensitive to motion artifacts. The absorption coefficient of whole blood is relatively higher at ∼400−600  nm than beyond 650 nm.^36^ The scattering coefficient of tissue also decreases beyond 650 nm.^10^ A decrease in blood absorption and scattering results in higher light penetration into the tissue for longer wavelengths of light.^40^ Therefore, at longer wavelengths, the relative contribution from changes in Fresnel reflections (e.g., during motion artifacts) at the optical interfaces will be higher than from tissue backscatter in the SFS signal. This is supported by our simulation data where we saw light from 670-680 nm to be about 28 times less sensitive to blood absorption than that at 545 nm (Sec. 3.1).

Further, we used this 670 to 680 nm wavelength to quantify the relative degree of motion artifacts during two different experiments. This was achieved by calculating $\sigma_{\mathrm{DM}}$ as in Sec. 3.2.1. We performed MC simulations, which revealed that transmission-based changes indeed cause baseline shifts in the collected backscatter ($I_{b}^{M}$) and calculated reflectance ($R_{\mathrm{SF}}^{M}$). The RMSD calculations (Sec. 3.3) show similarity in the magnitude of shifts between simulation and experiment. We further show in Sec. 3.3 that the extracted % ${\mathrm{sO}}_{2}$ is relatively immune to such artifacts in $R_{\mathrm{SF}}^{M}$ due to its dependence on the shape of the reflectance spectrum.^38^ However, perfusion measurements are susceptible to these artifacts. A mathematical model for light transmission in the SFS is presented in Sec. 3.4 and extended to an MAC algorithm. During MAC, $I_{b\;\text{at}\;680\;\mathrm{nm}}^{M}$ is used to correct for motion artifacts using a linear regression-based method. The MAC algorithm was first validated using dynamic simulation data in Sec. 3.5. The advantage of the simulation is that it enables the computation of data without artifacts which is difficult to do with confidence during experiments. Simulations showed optimal performance by the 680 nm wavelength in reducing the artifactual changes. Further, we used mean square error (MSE) to evaluate MAC performance. While we observed a reduction of MSE after the correction of motion artifacts by two orders of magnitude, the minor variations observed in the MSE between Figs. 4(a)–4(c) in the artifact-free region-1 and region-3 can be attributed to the variations produced during MC simulations as supported in the supplementary data [Fig. S1(c) in the Supplementary Material]. To show the importance of selecting a hemodynamic insensitive wavelength, we have provided data where we test the MAC algorithm with an arbitrarily picked 590 nm wavelength. Results show suboptimal performance for MAC using the 590 nm wavelength due to its increased sensitivity to blood absorption compared to that of 680 nm (Fig. S3 in the Supplementary Material).

Testing the MAC algorithm using *in-vivo* data showed a dramatic reduction of motion artifact-based changes as seen comparing Figs. 2(b) and 5(a). The DM [e.g., as seen by higher $\sigma_{\mathrm{DM}}$ in Fig. 2(f)] showed a higher variability prior to correction, which was minimized post-correction as seen in Figs. 5(c) and 5(e). While the MAC algorithm was successful in reducing the variation in the signals, it also appears that it reduces the perfusion signal [Figs. 5(d) and 5(f)]. Prior to the correction, the perfusion during pre-stim condition was significantly lower than that during stim [Fig. 2(g)], but the relative contributions of hemodynamics and motion artifacts to this change were unclear. After correction, there is still a significant difference [Figs. 5(d) and 5(f)] which can be attributed substantially to hemodynamics as motion artifact-based changes are minimized by the MAC algorithm. The corrected $I_{b}^{M}$ trace in Fig. 5(a) shows slow changes, which are characteristic of hemodynamic signals.

### 4.1 Limitations

There are some limitations to this study. Firstly, the differences in the hemodynamic features between simulations [Fig. 3(a)] and experiment [Fig. 2(d)] can be attributed to the approximations used in the MC simulation to model the *in-vivo* situation. For example, tissue was assumed to be homogeneous and the data to estimate the various MC model parameters (e.g., BV, scattering, and absorption properties) from deep brain regions is limited, which can result in the observed differences in the hemodynamic features. Future studies could be aimed at identifying more reliable estimates of various MC model parameters for SFS-based measurements.

Second, the small sample size of three animals per experiment, is a limitation of our study. However, to emphasize results, we performed statistical tests on the data by partitioning it as described in Secs. 3.2.1 and 3.6. A similar approach has been used previously to compare peak versus baseline ${\mathrm{Ca}}^{2+}$ activity in the PVN.^22^ The Wilcoxon sign test used to test significance in Figs. 2(f)–2(g), Figs. 5(c), and 5(e)–5(f) models the different samples as independent. This assumption is reasonable in our data due to the random and uncorrelated nature of the variations due to motion artifacts in the data.

Thirdly, due to the non-zero sensitivity of 680 nm light to hemodynamics, the MAC process leads to some attenuation of the corrected trace. For example, in Fig. 5(d), we observe that the raw trace shows a larger perfusion level than the corrected trace. The faster return to the baseline of the corrected trace than the raw trace can also be attributed to the correction. More studies will be required to study the difference in the temporal features between the corrected and raw traces. While our setup was limited to significant light (>10% of maximum) from 425 to 710 nm, other wavelengths may work better. Future work aims to identify further longer wavelengths in the near-infrared regions, which have lower sensitivity to hemodynamics than 680 nm to further improve the MAC process. Nevertheless, the methods presented in this paper can be used to visualize and analyze corrected signals, provided we apply identical MAC to all signals being analyzed. Certain spike-like artifacts that were present in Fig. 2(b) are minimized but not completely removed after applying the MAC algorithm. Testing revealed that these abrupt spikes in the $I_{b}^{M}$ post-MAC are due to outliers limiting the regression-based MAC to minimize such spikes. Future work can investigate constrained optimization using a smoothing cost function and interpolation-based smoothing as an additional step for fine-tuning the processed signal after applying the MAC algorithm described in Sec. 3.4.

## 5 Conclusions

In conclusion, we found that the wavelength region from 670 to 680 nm of the reflectance data is a good indicator for the amount of motion artifacts present in the hemodynamic signals acquired by the SFS. We observed that the $\sigma_{\mathrm{DM}}$ metric can be used to effectively represent changes in the acquired signals during episodes of large speculated motion. This work, as supported with the literature reveals the fact that the motion artifacts can be modeled as transmission changes in the illumination/collection path of light. Such transmission changes can cause shifts in the reflectance data in the experiment. This observation is in agreement with the MC simulations where we varied the transmission in the optical path of light. Secondly, the mathematical modeling of light intensity has been done at various optical interfaces during light propagation through the optical fiber. This framework was extended to a MAC algorithm. The MAC algorithm has been developed and tested using data generated from MC simulations. It is observed that the MAC can minimize motion artifact-based changes while conserving the stimulus-evoked hemodynamic change. Finally, we have tested the MAC algorithm on experimental data where we observed a decrease in the $\sigma_{\mathrm{DM}}$ metric post-correction augmenting the importance of the proposed MAC algorithm. We envisage that the methods to address and account for motion artifacts that have been presented in this work will act as stepping stone for future studies dealing with motion artifacts for SFS and other optical probe-based modalities.

## Supplementary Material



## Data Availability

Details about materials and methods are given in the paper. All code and data in support of the findings of this paper are available at https://figshare.com/s/9f0fe4b39272bb315a87.
