# Supplementary material for: Distinguishing motion artifacts during optical fiber-based in-vivo hemodynamics recordings from brain regions of freely moving rodents
Source: Neurophotonics. 2024 May 24;11(Suppl 1):S11511. doi: 10.1117/1.NPh.11.S1.S11511 (PMC11123205; doi:10.1117/1.NPh.11.S1.S11511)
Supplement: Supplementary file 1 [file NPh_011_S11511_SD001.pdf]

Supplementary information

1. Justification of 10^6 number of photons used during MC simulation.

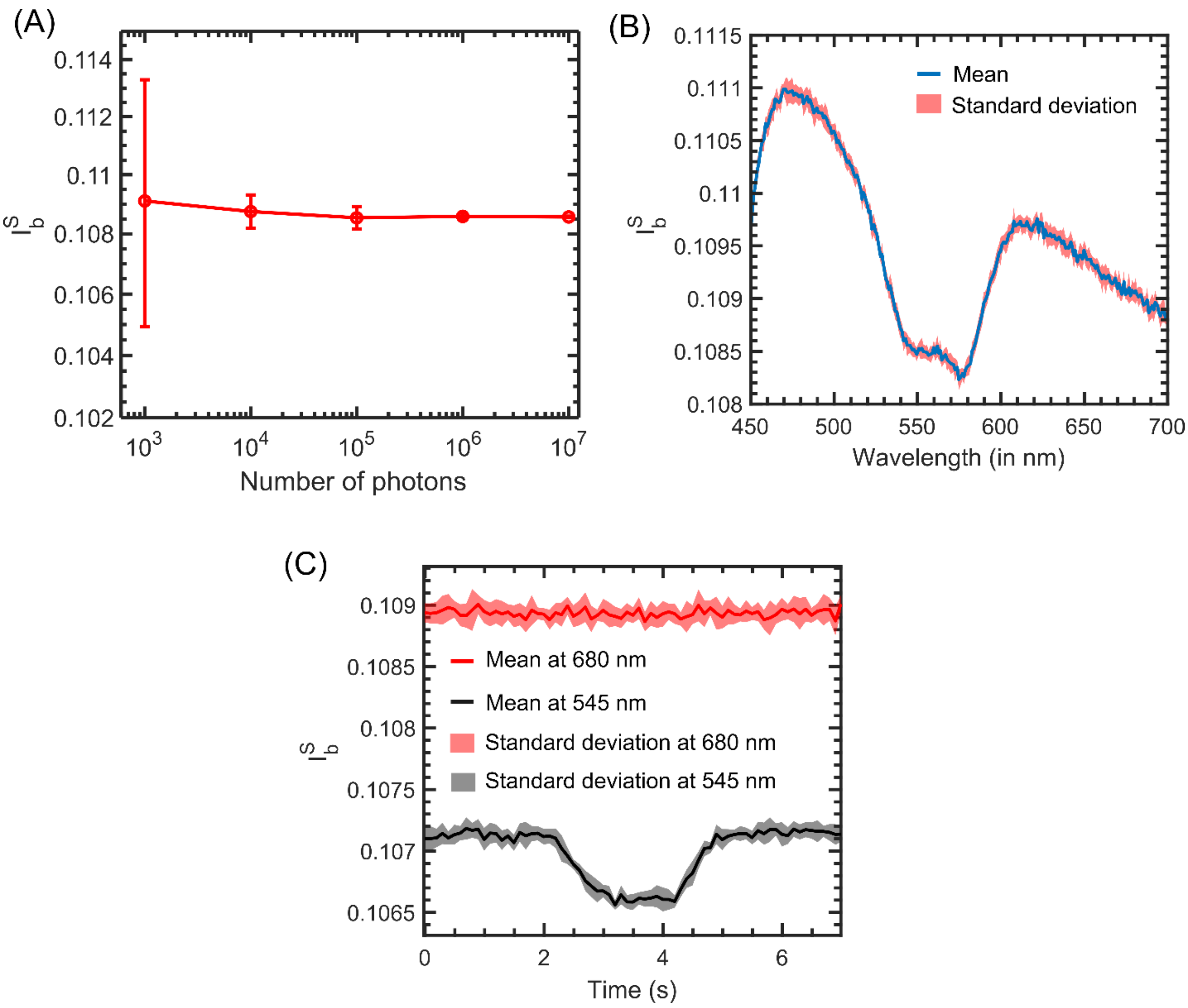

Fig. S1: (A) Shows the mean $\pm$ standard deviation across 10 simulation runs of I_b^S at 545 nm for 10^3,10^4, 10^5, 10^6, 10^7 photons. (B) Shows the mean $\pm$ standard deviation across 10 simulation runs of I_b^S for 450-700 nm for 10^6 photons for 35 % sO_2, 1 % blood volume, and nominal transmission at the far end. (C) Shows the mean $\pm$ standard deviation across 10 simulation runs of stimulus-evoked I_b^S at 545 nm and 680 nm wavelength for 10^6 photons.

We calculated the mean and standard deviation of I_b^S across 10 MC simulations for 545 nm wavelength to evaluate the variation in I_b^S as a function of number of photons used during the MC simulations. The tissue properties during this set of simulations were set to simulate 35 % sO_2 and 1 % blood volume (BV). The results for different number of photons used is shown in Fig S1(A). We observe that the simulated values have a lower standard deviation with increasing number of photons which is a well-known process observed in MC simulations for photon propagation in the tissue. Further we observed a small difference in the coefficient of variation (CoV) of 0.1 % and 0.02 % for the 10^6 and 10^7 photons used, respectively.

Fig. S1(B) shows the mean and standard deviation of I_b^S across 10 MC simulations for various wavelengths across the spectrum from 450-700 nm for 35 % sO_2, 1 % blood volume and nominal transmission (identical to data in Fig. 3 (A)). We observe that the hemodynamic absorption features between 540-590 nm are well over the standard deviation of R_{SF} observed at all wavelengths. Fig. S1(C) shows the mean and standard deviation of I_b^S across 10 MC simulations for I_b^S generated using details in Sec. 2.3.2 for 545 nm and 680 nm during absence of motion artifacts. We observe that the 0.52 % hemodynamic change observed during stimulus was larger than 0.1 % CoV observed with 10^6 photons.

Together, these data indicate that using 10^6 photons in an MC simulation is sufficient for the conclusions we draw.

2. Details for simulating stimulus evoked changes using MC simulations.

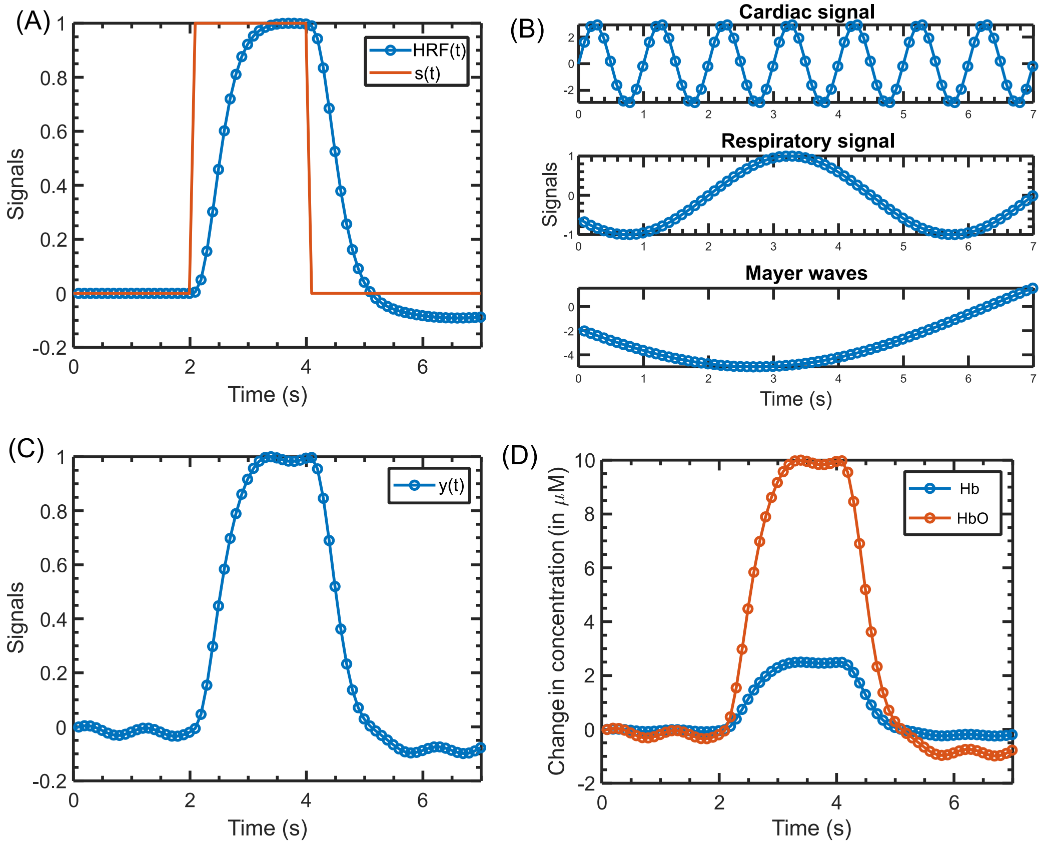

Fig S2. (A) shows the stimulus function (s(t)) and the hemodynamic response function ($HRF(t)$). (B) shows the different physiological frequencies used during the simulations. The plots represent $a_c \sin(2\pi f_c k)$, $a_r \sin(2\pi f_r k)$, $a_m \sin(2\pi f_m k)$, where $a_c = 3$, $a_r = 1$ and $a_m = 5$ and $f_c = 1$ Hz, $f_r = 0.2$ Hz and $f_m = 0.07$ Hz where c, r, m represent cardiac signal, respiratory signal and Mayer waves (C) shows the plot for y(t) which is obtained by normalizing $R(t)$ described in Eq. (4). (D) shows the change in hemoglobin concentrations created by appropriately scaling $y(t)$ as $\Delta C_{HbO} \cdot y(t)$ and $\Delta C_{Hb} \cdot y(t)$ for oxyhemoglobin and deoxyhemoglobin, respectively. (C). This is used in Eq. (5) to generate changes in tissue absorption.

3. Testing the MAC algorithm using 590 nm instead of 680 nm

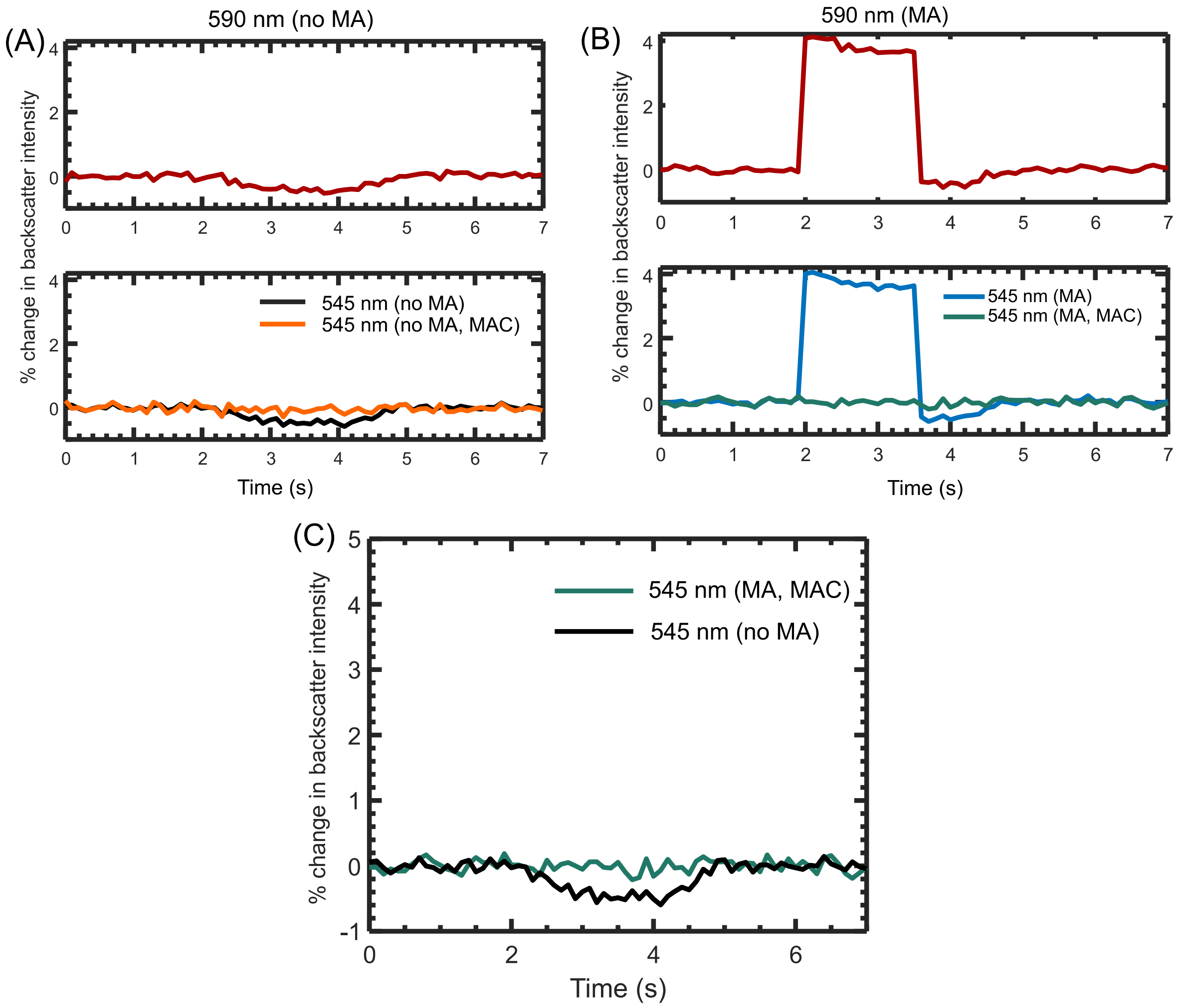

Fig S3. Simulations showing % changes in backscatter intensity from a single run for (A) at 590 nm, 545 nm without motion artifact (MA) and corresponding corrected 545 nm data using MAC algorithm. (B) shows data at 590 nm during motion artifact, 545 nm during motion artifact and corresponding corrected data at 545 nm using MAC algorithm. (C) shows comparison of 545 nm data processed by MAC algorithm and 545 nm without motion artifact.

Here we recreate Fig. 4 but by using 590 nm for MAC instead of the 680 nm wavelength. In contrast to Fig. 4(A), we observe that the MAC process using 590 nm leads to drastic reduction in the hemodynamic change in Fig. S3 (A) which is the case without motion artifacts (MA). Similarly in Fig. S3(B), we are not able to recover the change in perfusion which was possible in Fig. 4 (B). Compared to Fig. 4(C), the Fig. S3(C) further shows that the corrected 545 nm trace (545 nm MA, MAC) is not able to closely follow the ideal 545 nm trace (i.e., 545 nm, no MA). The suboptimal performance observed during MAC using 590 nm is due to relatively large absorption of light at this wavelength due to hemoglobin than 680 nm. While the attenuation of the signal is an inherent limitation of the correction process presented in this paper, the objective is to trade-off between the reduction of motion artifacts and the attenuation of the signal which we can achieve. Since 590 nm is more sensitive to hemodynamics, 680 nm is a better choice.
